# Comparing the genetic architecture of childhood behavioral problems across socioeconomic strata in the Netherlands and the United Kingdom

**DOI:** 10.1007/s00787-019-01357-x

**Published:** 2019-06-01

**Authors:** A. M. Hendriks, C. Finkenauer, M. G. Nivard, C. E. M. Van Beijsterveldt, R. J. Plomin, D. I. Boomsma, M. Bartels

**Affiliations:** 1 Vrije Universiteit Amsterdam, Department of Biological Psychology, Netherlands Twin Register, van der Boechorststraat 7, 1081 BT Amsterdam, the Netherlands; 2 Amsterdam Public Health Research Institute, Amsterdam, the Netherlands; 3 Department of Interdisciplinary Social Sciences: Youth Studies, Utrecht University, Padualaan 14, 3584 CH Utrecht, the Netherlands; 4 King’s College London, Social, Genetic and Developmental Psychiatry Centre, MRC Social, Institute of Psychiatry, Strand, London, WC2R 2LS UK

**Keywords:** Childhood behavioral problems, Socioeconomic status, Netherlands, UK

## Abstract

Socioeconomic status (SES) affects the development of childhood behavioral problems. It has been frequently observed that children from low SES background tend to show more behavioral problems. There also is some evidence that SES has a moderating effect on the causes of individual differences in childhood behavioral problems, with lower heritability estimates and a stronger contribution of environmental factors in low SES groups. The aim of the present study was to examine whether the genetic architecture of childhood behavioral problems suggests the presence of protective and/or harmful effects across socioeconomic strata, in two countries with different levels of socioeconomic disparity: the Netherlands and the United Kingdom. We analyzed data from 7-year-old twins from the Netherlands Twin Register (*N* = 24,112 twins) and the Twins Early Development Study (*N* = 19,644 twins). The results revealed a nonlinear moderation effect of SES on the contribution of genetic and environmental factors to individual differences in childhood behavioral problems. The heritability was higher, the contribution of the shared environment was lower, and the contribution of the nonshared environment was higher, for children from high SES families, compared to children from low or medium SES families. The pattern was similar for Dutch and UK families. We discuss the importance of these findings for prevention and intervention goals.

## Introduction

Childhood behavioral problems comprise of aggressive and non-aggressive behaviors such as fighting, lying, stealing, or disobedience [1, 2]. Childhood behavioral problems co-occur with many other symptoms of childhood psychopathology such as hyperactivity, inattention, and anxiety [3]. Moreover, they are considered a potential marker for psychopathology in later life [4, 5]. Childhood behavioral problems are, in addition, associated with substantial costs for families and society [6, 7]. Given its profound effects for healthy development, it is important to gain more insight into risk factors for childhood behavioral problems.

One factor that is consistently found to be associated with childhood behavioral problems is socioeconomic status (SES), with children from low SES families displaying more behavioral problems than children from high SES families [8, 9]. SES refers to indicators of people’s standing in the stratification system and is usually measured by education, occupation, employment, income, and/or wealth. SES determines a families’ access to social, material, or cultural resources, for example, as a result of the parents’ educational qualifications [10, 11].

Despite evidence for an overall association between childhood behavioral problems and SES, an underexplored question in the field is whether differences in childhood behavioral problems emerge due to different underlying genetic and environmental processes for children from low SES families compared to children from high SES families. A powerful approach to gain more insight in the etiology of childhood behavioral problems is through twin studies. Twin studies have the ability to discern the contribution of genetic factors, shared environmental factors, and nonshared environmental factors to differences in childhood behavioral problems [12]. So far, twin studies have demonstrated that genetic differences between children explain about 52–78% of the differences in parent-reported behavioral problems in children. Shared environmental factors account for about 1–36% of the differences in childhood behavioral problems [13–19]. In addition, twin studies have shown that the contribution of genetic and environmental factors to individual differences in childhood behavioral problems varies across SES strata, indicating a moderator effect of SES on the genetic architecture of childhood behavioral problems. For example, research in children from the Netherlands [20] and research on adolescents from Sweden [21] report lower heritability, higher influence of the shared environment, and lower influence of the nonshared environment on behavioral problems in children from lower SES families compared to children from higher SES families. These findings suggest that the influence of shared environmental factors is amplified in disadvantageous environments, such as low SES, or reduced by advantageous environments.

The literature typically assumes that the effect of SES on both means and variances is linear, such that children with lower SES show more behavioral problems than children with higher SES or that the heritability is lower in low SES groups than in high SES groups, respectively. Alternatively, there may be separate protective and harmful effects, which can be examined using a trichotomization approach [22]. This approach compares the mid-range of a variable’s distribution, in our case SES, with the upper and lower ends. An effect limited to low SES would be distinguishable if, for example, only children with low SES were to show more behavioral problems than the children in the mid-range and high SES. Put differently, children with mid-range and high SES would not differ in behavioral problems, but would show less behavioral problems than those with low SES, indicating that low SES is a risk factor for childhood behavioral problems. Furthermore, trichotomization of variance components could distinguish shifts in heritability (or a common environment) due to protective effects from shifts in heritability due to harmful effects. Gaining knowledge about the SES conditions that are promotive versus risky for the development of childhood behavioral problems is critical to our understanding of factors associated with childhood behavioral problems and can be used to tailor interventions to fit the needs of children from different social strata.

To get a better hold of the possible effects of SES on levels and variation of behavioral problems in middle childhood we, in the current study, compare two countries with different levels of socioeconomic disparity; the Netherlands, and the United Kingdom [23]. While the contribution of genetic and environmental factors to individual differences in childhood behavioral problems appears to be similar in the Netherlands and the United Kingdom [18], comparability of effects of SES on the variance decomposition is unknown. Because of the larger difference between low and high SES in the United Kingdom compared to the Netherlands [23], we expect larger differences in the etiology of childhood behavioral problems (e.g., the contribution of genetic and environmental factors to individual differences in childhood behavioral problems) across SES strata in the United Kingdom than in the Netherlands.

The present study aims to (1) investigate linearity of the moderating effect of SES on the genetic architecture of childhood behavioral problems and (2) investigate whether the moderating effect of SES differs between the Netherlands and the United Kingdom. To this end, we analyzed twin data from two large longitudinal prospective twin cohorts in the two countries. To allow for nonlinear effects of SES, we categorized SES into three strata.

## Method

### Participants and measures

#### Data from the Netherlands

The Netherlands twin register (NTR) is a nation-wide population-based register founded in 1987 in the Netherlands [24]. For the present study, we included mother-reported data for 7-year-old twins (*N* = 24,826) born between 1986 and 2006. We excluded twin pairs in which one or both twins had a disease or disability that interfered with daily functioning (*N* = 714). The final sample consisted of 12,056 twin pairs (*N* = 24,112 twins, *M* age = 7.45 years, *SD* = 0.40, 49.7% males). Socioeconomic status (SES) was based on parental level of education. Based on the highest educational qualification of either the mother or the father assessed at age seven, we categorized children’s SES as low, medium, or high.

Behavioral problems were assessed using maternal ratings on the Aggressive Behavior syndrome subscale of the Child Behavior Checklist (CBCL) [1]. This scale consisted of 18 items that assessed aggressive and non-aggressive behaviors such as “Disobedient at home”, “Gets in many fights”, and “Sulks a lot”. Mothers were asked to report on their child’s behavior in the past 6 months. Response categories were: 0 = “Not true”, 1 = “Sometimes or somewhat true”, and 2 = “Very true or often true”. If more than three items were missing, participants were not included; otherwise, the mean score was imputed for missing items.

#### Data from the United Kingdom

The Twins early development study (TEDS) is a twin register that longitudinally follows the development of twins born between 1994 and 1996 in England and Wales (from here on referred to as the United Kingdom) [25]. For the present study, we included parent-reported data for 7-year-old twins (*N* = 20,685). We excluded 515 twin pairs in which one or both twins had a disease or disability that interfered with daily functioning. The final sample consisted of 9,822 twin pairs (*N* = 19,644 twins, *M* age = 7.07, *SD* = 0.25, 48.7% males). Like in the NTR, SES was based on parental level of education for the TEDS sample. Based on the highest educational qualification of either the mother or the father, assessed at first contact, we categorized children’s SES as low, medium, or high. Although the educational system differs between the Netherlands and the United Kingdom, we established comparable categories, as displayed in Table 1.

**Table 1:** SES categories

SES	The Netherlands	The United Kingdom
Low	Elementary school Few years of more extensive primary education (mulo) Graduated mulo or general secondary education (mavo) Few years of lower technical education (lts) Few years higher general secondary education (havo)/pre-university education (vwo)	No qualifications CSE grade 2–5 or 0-level/GCSE grade D-G
Medium	Graduated havo/vwo Few years intermediate vocational education (mbo) Graduated mbo Few years of higher vocational education (hbo) or university	CSE grade 1 or 0-level/GCSE grade A-C A-level or S-level Higher National Certificates
High	Graduated hbo Graduated university Postgraduate	Higher National Diplomas Undergraduate Postgraduate qualification

Behavioral problems were assessed using parental (97.3% maternal ratings) ratings on the Conduct Problem subscale of the Strengths and Difficulties Questionnaire (SDQ) [2]. This scale consisted of five items that assessed aggressive and non-aggressive behaviors such as “Often fights with other children or bullies them”, “Generally obedient, usually does what parents request”, and “Often has temper tantrums or hot tempers”. Parents were asked to report on their child’s behavior. Response categories were: 0 = “Not true”, 1 = “Somewhat true”, and 2 = “Certainly true”. If more than two items were missing, participants were not included; otherwise, the mean score was imputed for missing items.

### Statistical analysis

To gain insight in the distribution of childhood behavioral problems across sex, SES, and countries, we obtained descriptive statistics using R. Next, we performed twin analyses in R (version 3.4.3) with the package OpenMx (version 2.8.3) with the NPSOL optimizer [26].

### Twin analyses

With twin models, by comparing resemblance on a trait between monozygotic (MZ) and dizygotic (DZ) twins, it is possible to disentangle to which extent individual differences in a trait can be explained by genetic variance (A), variance due to the shared environment (C), and variance due to the nonshared environment (E) [12]. We extended the model by including two moderators to test whether the contribution of genetic and environmental variance to individual differences in childhood behavioral problems interacted with these moderators (i.e., SES strata and country).

Because the distribution of childhood behavioral problems was highly skewed, we categorized the variable by applying two thresholds, partitioning the sample in the 33% lowest scoring, the middle 33%, and 33% highest scoring children on childhood behavioral problems. This method has the advantage of optimizing parameter estimates [27]. We fitted the thresholds for the Netherlands and the United Kingdom separately, before entering them into the model. To simultaneously compare SES strata and countries, we fitted a 30 group model containing all groups (e.g., MZ male, DZ male, MZ female, DZ female, DZ opposite sex × SES low, SES medium, SES high × the Netherlands and the United Kingdom). Categorizing SES into low, medium, and high allowed us to test for both protective and negative moderating effects of (high/low) SES. Because studies so far did not find evidence for qualitative or quantitative sex differences for childhood behavioral problems [18, 28], we constrained the correlations and A, C, and E components to be equal for boys and girls and opposite-sex twin correlations to be equal to DZ correlations. To account for the frequently observed mean differences in behavioral problems, thresholds were allowed to vary across sex.

### Model fitting

We tested moderator effects by stepwise testing whether constraining parameters to be equal across moderator categories significantly deteriorated goodness of fit (i.e., *p* < 0.01). If a constraint did not significantly deteriorate model fit, we proceeded with applying this constraint in the later models. Based on the best fitting model, we estimated the influence of genetic and environmental factors on childhood behavioral problems across SES strata and countries.

First, we fitted a saturated model with thresholds freely estimated across sex, SES strata, and countries, and with correlations freely estimated across SES strata and countries. Next, we fitted the following models to test for the moderating effects of SES and country: thresholds constrained to be equal across SES strata; correlations constrained to be equal across SES strata; thresholds constrained to be equal across country; and correlations constrained to be equal across country.

Based on the findings from the saturated model, we specified the ACE model with the same 30 groups, constraining thresholds and A, C, and E in line with the results from the previous models to test the moderating effect of SES and country on the contribution of genetic and environmental factors to childhood behavioral problems. We first compared the ACE model to the best saturated model. Next, we tested the moderator effects by constraining A, C, and E across moderator categories.

For interpretational purposes, we performed additional analyses; we fitted the best ACE model but then with the thresholds constrained and the means and variances freely estimated. This model allowed us to examine the absolute variance of childhood behavioral problems across SES strata and countries. Based on this model, we reported the absolute values of A, C, and E.

## Results

The descriptives in Table 2 reveal that means were higher for boys than girls, behavioral problems decreased as SES increased, and the variance decreased as SES increased. The patterns were similar between the Netherlands and the United Kingdom. The twin correlations, as displayed in Table 3, suggest that for all three SES strata, genetic factors played a role, because the MZ correlations were higher than the DZ correlations. Because the DZ correlations were larger than half of the MZ correlations, we suspected shared environmental effects. MZ and DZ twin correlations were highest for low SES, were slightly lower for medium SES, and lowest for high SES, suggesting an increased contribution of the nonshared environment for higher SES; the difference between MZ and DZ correlations was constant across SES strata. These patterns occurred both in the Netherlands and in the United Kingdom.

**Table 2:** Descriptive statistics

SES strata	N pairs	Mean boys	Variance boys	Mean girls	Variance girls	Total mean	Total variance
The Netherlands							
SES low	2109	6.64	31.08	5.29	23.16	5.96	27.55
SES medium	5143	6.07	28.20	4.68	20.52	5.37	24.77
SES high	4730	5.04	24.57	3.75	14.68	4.40	20.05
Total	12,046	5.77	27.74	4.43	19.08	5.09	23.83
The United Kingdom							
SES low	1372	2.59	3.91	2.06	3.13	2.27	3.49
SES medium	5059	1.96	3.13	1.60	2.33	1.74	2.66
SES high	3265	1.61	2.40	1.24	1.82	1.40	2.08
Total	9822	1.88	2.98	1.51	2.29	1.69	2.66

**Table 3:** Twin correlations

SES strata	The Netherlands		The United Kingdom	
	MZ	DZ	MZ	DZ
SES low	0.87 [0.84, 0.89]	0.55 [0.49, 0.60]	0.83 [0.76, 0.88]	0.53 [0.43, 0.62]
SES medium	0.85 [0.83, 0.87]	0.53 [0.49, 0.57]	0.79 [0.75, 0.82]	0.49 [0.43, 0.54]
SES high	0.76 [0.73, 0.79]	0.41 [0.36, 0.46]	0.70 [0.64, 0.76]	0.41 [0.34, 0.48]

The results of the model fitting are displayed in the upper half of Table 4. We fitted a saturated model allowing for sex differences on the thresholds, but with correlations constrained to be equal for boys and girls (model 0). To test for the moderating effect of SES on childhood behavioral problems, we first constrained thresholds to be equal across SES strata (model 1). Applying this constraint significantly deteriorated model fit, indicating that the prevalence of behavioral problems varied over SES strata, with higher liability for children of low SES families to be in the group scoring highest on behavioral problems in both countries. Next, we constrained twin correlations to be equal across SES strata (model 2). This constraint significantly decreased model fit, indicating that there were SES effects on the correlations. MZ and DZ correlations were highest for low SES, slightly lower for medium SES, and lowest for high SES, with the difference between MZ and DZ remaining constant, suggesting that the contribution of genetic factors increased, shared environment decreased, and the nonshared environment increased for higher SES. To test the moderating effect of country on childhood behavioral problems, we constrained thresholds to be equal across countries (model 3). This constraint significantly deteriorated model fit, indicating that the thresholds were significantly different between countries. This difference indicated that the liability of children to fall within the lowest, middle, or highest scoring group of behavioral problems was different between the Netherlands and the United Kingdom. The first threshold was lower for the Netherlands, the second threshold was only lower in the Netherlands for boys from medium or high SES, the other second thresholds were higher in the Netherlands. This indicated a higher liability to belong to the middle scoring group of behavioral problems, but a lower liability to belong to the highest scoring group for boys and for girls from low SES for children in the Netherlands compared to children in the United Kingdom. Next, we constrained correlations to be equal across countries (model 4). This constraint did not decrease model fit, indicating that there were no effects of country on the correlations. Table 5 displays the parameter estimates of the final saturated model (model 4).

**Table 4:** Statistics of the fitted models

Model number	Model	Estimated parameters	− 2 LL	*df*	AIC	Compared to model	Δ − 2 LL	Δ *df*	*p*
*Saturated model*									
0	Saturated model with thresholds equal for MZ and DZ twins, correlations equal for boys and girls	36	61,852.35	30,693	466.35				
1	Thresholds equal across SES	20	62,132.26	30,709	714.26	0	279.91	16	3.63e−50
2	Correlations equal across SES	28	61,915.42	30,701	513.42	0	63.07	8	1.16e−10
3	Thresholds equal across countries	24	62,155.55	30,705	745.55	0	303.19	12	1.00e−57
4	Correlations equal across countries	30	61,865.90	30699	467.90	0	13.54	6	0.04
*ACE model*									
5	ACE model based on the results of the final saturated model	33	62,175.85	30,696	783.85				
6	A, C, and E equal across low, medium, and high SES	27	62,234.37	30,702	830.37	5	58.52	6	9.01e−11
6.1	A, C, and E equal across low and medium SES	30	62,179.76	30,699	781.76	5	3.91	3	0.27
6.2	A, C, and E equal across low and high SES	30	62,218.81	30,699	820.81	5	42.96	3	2.51e−09
6.3	A, C, and E equal across medium and high SES	30	62,215.38	30,699	817.38	5	39.53	3	1.34e−08

Model number corresponds to the description in “Results”. − 2 LL refers to the minus 2 log likelihood. df represents the number of degrees of freedom of the model. Δ − 2 LL refers to the difference in minus 2 log likelihood of the compared models. Δ df refers to the difference in number of degrees of freedom of the compared models. p corresponds to the *p* value of the difference in model fit between the compared models

**Table 5:** Parameter estimates of the best fitting saturated model (model 3) with 95% confidence intervals

	The Netherlands		The United Kingdom	
	Correlation		Correlation	
	MZ	DZ	MZ	DZ
SES low	0.86 [0.83, 0.88]	0.54 [0.49, 0.59]	0.86 [0.83, 0.88]	0.54 [0.49, 0.59]
SES medium	0.83 [0.81, 0.85]	0.51 [0.48, 0.54]	0.83 [0.81, 0.85]	0.51 [0.48, 0.54]
SES high	0.74 [0.71, 0.77]	0.41 [0.37, 0.45]	0.74 [0.71, 0.77]	0.41 [0.37, 0.45]

		Mean	Variance	Mean	Variance
SES low	Boys	− 0.86 [− 0.93, − 0.79]	0.09 [0.03, 0.16]	− 0.67 [− 0.78, − 0.57]	0.01 [− 0.09, 0.11]
	Girls	− 0.64 [− 0.71, − 0.57]	0.35 [0.28, 0.41]	− 0.45 [− 0.55, − 0.35]	0.21 [0.11, 0.30]
SES medium	Boys	− 0.75 [− 0.80, − 0.71]	0.19 [0.15, 0.24]	− 0.49 [− 0.54, − 0.43]	0.27 [0.22, 0.32]
	Girls	− 0.55 [− 0.59, − 0.51]	0.49 [0.44, 0.53]	− 0.29 [− 0.35, 0.24]	0.48 [0.42, 0.53]
SES high	Boys	− 0.60 [− 0.64, − 0.55]	0.41 [0.37, 0.45]	− 0.26 [− 0.33, − 0.20]	0.48 [0.41, 0.54]
	Girls	− 0.37 [− 0.41, − 0.32]	0.69 [0.65, 0.74]	− 0.09 [− 0.16, − 0.03]	0.65 [0.58, 0.71]

Based on the outcomes from the saturated model (model 4), we fitted the ACE model allowing thresholds to vary across sex, SES strata, and countries, and allowing A, C, and E to vary across SES strata (model 5). Because in the final saturated model, the MZ and DZ correlations did not differ significantly between the Netherlands and the United Kingdom, we constrained A, C, and E to be equal across country. The results of the ACE model fitting are displayed in the lower half of Table 4. We tested the moderating effect of SES by constraining A, C, and E to be equal across SES strata (model 6). This constraint significantly deteriorated model fit, indicating that the values of A, C, and E were significantly different for low, medium, and high SES. Between low and medium SES, A, C, and E were distributed similarly. For high SES compared to low and medium SES, A appeared to be higher, C lower, and E higher. To explore the moderating effect of SES, we performed pairwise comparisons on the A, C, and E estimates between SES strata to test which strata significantly differed from each other. The difference between low and medium SES (model 6.1) did not significantly decrease model fit. The difference between low and high SES (model 6.2), and medium and high SES (model 6.3) did significantly decrease model fit, indicating a significant difference in A, C, and E between low and high SES and medium and high SES. A appeared to be higher, C lower, and E higher, for high SES compared to both low SES and medium SES.

**Fig. 1:**
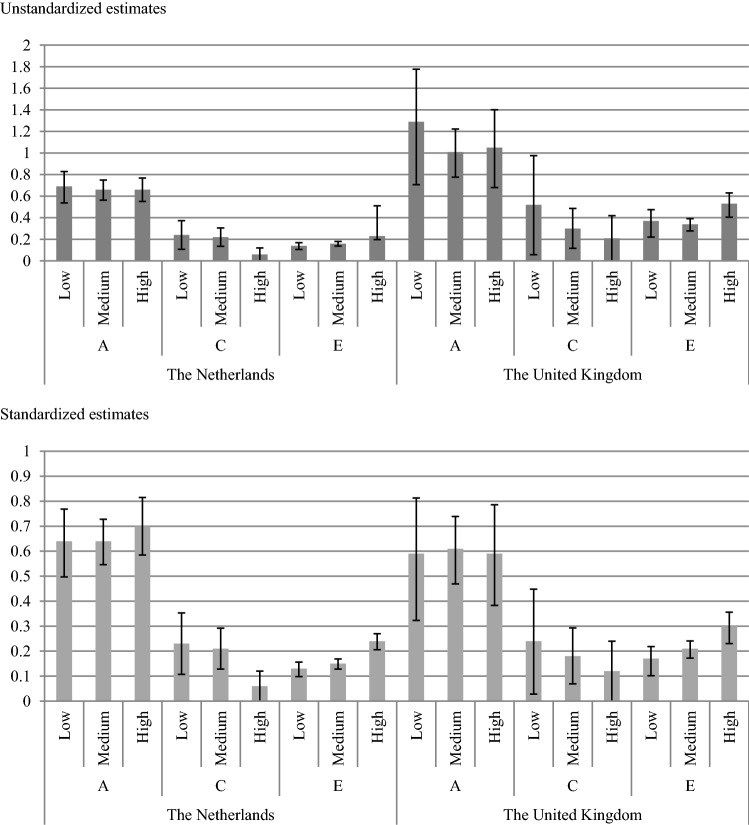
Unstandardized and standardized estimates of variance components from the best fitting ACE model with 95% confidence intervals. The top half of the figure represents unstandardized estimates of variance components due to A (additive genetic factors), C (shared environment), and E (nonshared environment) for the different SES strata (i.e., low, medium, and high) for the Netherlands on the left and the United Kingdom on the right, respectively. The lower half of the figure displays the standardized estimates of variance due to A, C, and E across SES strata and countries

The additional analyses revealed absolute differences in variance across SES (Δ − 2 LL = 13.58, Δ *df* = 4, *p* < 0.01), with the highest variance for low SES, lower variance for medium SES, and lowest variance for high SES. Furthermore, the absolute variance varied between the Netherlands and the United Kingdom (Δ − 2 LL = 221.37, Δ *df* = 3, *p* < 0.01); the variance was larger in the United Kingdom. Table 6 presents the absolute and standardized estimates across countries and SES strata. Figure 1 graphically displays the unstandardized and standardized parameter estimates. The parameter estimates and their confidence intervals suggested that the moderating effect of SES was mainly driven by E. Constraining A and C to be equal across SES strata, however, revealed that there were significant differences in A and C across SES strata (Δ − 2 LL = 32.92, Δ *df* = 8, *p* < 0.01).

**Table 6:** Parameter estimates of the best fitting ACE model (model 4) with 95% confidence intervals from the model estimating the thresholds and separately for the Netherlands and the United Kingdom from the model estimating the absolute variance

SES category	A unstandardized	C unstandardized	E unstandardized	A	C	E
NL SES low	0.69 [0.55, 0.84]	0.24 [0.11, 0.37]	0.14 [0.11, 0.18]	0.64 [0.51, 0.79]	0.23 [0.12, 0.35]	0.13 [0.11, 0.16]
NL SES medium	0.66 [0.57, 0.75]	0.22 [0.13, 0.30]	0.16 [0.14, 0.18]	0.64 [0.55, 0.73]	0.21 [0.13, 0.29]	0.15 [0.13, 0.18]
NL SES high	0.66 [0.55, 0.77]	0.06 [0.00, 0.15]	0.23 [0.20, 0.26]	0.70 [0.59, 0.82]	0.06 [0.00, 0.16]	0.24 [0.21, 0.27]
UK SES low	1.29 [0.81, 1.88]	0.52 [0.06, 0.98]	0.37 [0.27, 0.52]	0.59 [0.37, 0.86]	0.24 [0.03, 0.45]	0.17 [0.12, 0.24]
UK SES medium	1.01 [0.79, 1.24]	0.30 [0.12, 0.49]	0.34 [0.29, 0.41]	0.61 [0.48, 0.75]	0.18 [0.07, 0.30]	0.21 [0.18, 0.25]
UK SES high	1.05 [0.70, 1.42]	0.21 [0.00, 0.49]	0.53 [0.43, 0.66]	0.59 [0.39, 0.79]	0.12 [0.00, 0.27]	0.30 [0.24, 0.37]

## Discussion

The purpose of the present study was to investigate whether the genetic architecture of childhood behavioral problems differs across low, medium, and high SES. In addition, we examined whether this effect varied between the Netherlands and the United Kingdom, two countries that differ in income disparity [23]. For this, we used data from two large longitudinal prospective twin cohorts. We found more childhood behavioral problems in children from low SES compared to medium SES, and from medium SES compared to high SES. Furthermore, children from the Netherlands were more likely to be in the middle scoring group on childhood behavioral problems compared to children from the United Kingdom, whereas children from the United Kingdom were more likely to be in the high scoring group compared to children from the Netherlands, except for girls from medium and high SES. The variance of childhood behavioral problems was higher in the United Kingdom compared to the Netherlands.

In our study, SES did have a moderating effect on the contribution of genetic and environmental factors to childhood behavioral problems. Because we categorized SES into three strata, we were able to examine whether the moderating effect of SES on the genetic architecture of childhood behavioral problems may be due to the presence of advantageous factors (i.e., high vs. medium SES) or to the presence of disadvantageous factors (i.e., low vs. medium SES) [22]. The difference in the contribution of genetic and environmental factors to childhood behavioral problems between low and medium SES was not significant, while the difference between low and high SES and the difference between medium and high SES were significant. These findings indicate that the moderating effect of SES appears to be due to growing up in a more advantageous environment. Children from high SES families tend to have less exposure to environmental factors that increase the risk of behavioral problems (e.g., parental stress) and more exposure to environmental factors that may decrease the risk of behavioral problems (e.g., monitoring). The environment in high SES families may leave less room for individual differences in childhood behavioral problems, as evidenced in the non-significant contribution of the shared environment. Among children from high SES backgrounds, genetic and nonshared environmental factors appear to be more relevant in explaining variability in behavioral problems, compared to children from low or medium SES backgrounds, for whom the influence of the shared environment is larger. The total variance estimate was higher for low and medium SES, compared to high SES, indicating that children from low and medium SES were more heterogeneous in their levels of childhood behavioral problems. For the variance component estimates, the estimate of genetic variance was similar across SES strata, the estimate of the shared environment variance component was larger in low and medium SES than in high SES families, and the estimate of the nonshared environment variance component was higher for children from high SES families than for children from low and medium SES. This pattern occurred both in the Netherlands and in the United Kingdom. Our results were in line with the theory that under advantageous circumstances (i.e., high SES), genetic influences are more fully realized compared to less advantageous or disadvantageous circumstances in which genetic influences are more suppressed and the environment contributes more strongly [29].

A clinical implication from these results is that the etiology of childhood behavioral problems is different for children from low or medium SES families compared to children from high SES families. For children from low or medium SES families, the shared environment explains a larger proportion of the variance compared to children from high SES families, suggesting that these children could benefit from treatment aiming to ameliorate shared environmental factors [19]. Examples of these factors could be healthy family functioning, less parental stress, positive school attachment, or housing quality [22, 30–32].

We did not find evidence for our hypothesized stronger moderating effect of SES on the genetic architecture of behavioral problems for children in the United Kingdom than in the Netherlands. Nevertheless, this should be interpreted with caution, because the two countries measured behavioral problems with a different questionnaire. Porsch and colleagues [18] found that the contribution of genetic and environmental factors to childhood behavioral problems is similar for children from the Netherlands and the United Kingdom. Extending this finding, our results showed that the moderating effect of SES was comparable in the Netherlands and the United Kingdom. Although there is more income inequality in the United Kingdom compared to the Netherlands [23], the United Kingdom also, for example, invests a larger percentage of the Gross National Product in family benefits [33] and education [34], which may attenuate the effects of larger income inequality. A similar explanation is proposed for the different findings of the moderating effect of SES on the contribution of genetic and environmental factors to intelligence [35, 36]. Therefore, it would be promising for future research to investigate whether our findings replicate in other countries with different levels of income inequality and country investments in children and families.

### Strengths and weaknesses

One strength of this study is that we applied thresholds to take the skewed distribution of childhood behavioral problems into account. Studies so far on the moderating effect of SES on the genetic architecture of childhood behavioral problems included childhood behavioral problems as a continuous variable [20, 37]. Because of the non-normal distribution of childhood behavioral problems, analyzing behavioral problems continuously might lead to overestimated genetic variance and underestimated shared environmental variance. By incorporating thresholds, our analyses could have led to more precise parameter estimates [27]. Nevertheless, although we used a method to obtain more precise estimates compared to the previous articles, our results confirmed their findings regarding the moderating effect of SES on the contribution of genetic and environmental factors to individual differences in behavioral problems both in the Netherlands and the United Kingdom, indicating robustness. A second strength of this study is that we fitted all the estimates for the different SES strata and countries simultaneously, instead of separately for each country, allowing for formal comparison of parameter estimates across all groups.

Despite the strengths of our study, several limitations warrant mentioning. One limitation is that the measures of childhood behavioral problems differed between the Netherlands and the United Kingdom. For this reason, it is not certain whether the difference in means and variances between the Netherlands and the United Kingdom was due to true differences across countries or due to different measures. Furthermore, the different measures could lead to underestimation of the moderating effect of countries on the contribution of genetic and environmental factors to childhood behavioral problems. Although several studies found high comparability between the Child Behavior Checklist and the Strengths and Difficulties Questionnaire [38, 39], they assess different symptoms of behavioral problems. A recent study with data from the Netherlands and the United Kingdom found that the genetic architecture of childhood behavioral problems was similar for both instruments [18]. In this paper, we took a next step and allowed moderation of genetic architecture by SES. Here also, the outcomes suggested very similar findings for both instruments, but we acknowledge that we cannot state to which extent the results may be affected by different measures of behavioral problems and different countries. For example, whereas the sample from the United Kingdom represents the population [25], the sample from the Netherlands is on average more highly educated than the Dutch population [40]. Future research should investigate whether the genetic architecture of childhood behavioral problems varies across questionnaires in the same sample and whether our findings hold when employing the same questionnaire in the Netherlands and the United Kingdom.

A second limitation is that we used only parental education as measure of SES. Studies with TEDS data usually employ a measure of SES comprised of parental education, parental occupation, income, and sometimes maternal age at birth of the first child [41, 42]. However, because we used data from two different countries with different questionnaires, we decided to use a homogenous measure to optimize comparability between the data from the Netherlands and from the United Kingdom. An opportunity for future research would be to test whether our results regarding the moderating effect of SES on the genetic architecture of childhood behavioral problems across countries also apply to other measures of SES. Nevertheless, although the abovementioned limitations require more cautious interpretation of our findings, the present study does provide important insight in the genetic architecture of childhood behavioral problems across SES strata and countries.

A third limitation was that we only included a single age group. It is known that the contribution of genetic and environmental factors to individual differences in behavioral problems changes with age; the role of the shared environment disappears in adolescence [16, 43]. Furthermore, the way adolescents perceive their SES differs from how children perceive it [44]. In addition, the association between behavioral problems and SES decreases as children become older [9]. Therefore, it is likely that our findings cannot be generalized to other ages, and thus, it would be useful for future research to examine the moderating effect of SES on the genetic architecture of behavioral problems across childhood and adolescence.

## Conclusion

The present study sought to gain insight in the etiology of childhood behavioral problems by investigating whether the contribution of genetic and environmental factors varies across SES strata in the Netherlands and the United Kingdom. Our results showed that both in the Netherlands and the UK, shared environmental factors have a stronger effect in behavioral problems in children from low SES families, while genetic factors are most prominent for behavioral problems in children from medium and high SES families. These findings have important implications for prevention and intervention goals.
